# SleepBert: An Intelligent Clinical Encyclopaedia for Sleep Disorders Using Large Language Models

**DOI:** 10.21203/rs.3.rs-6605863/v1

**Published:** 2025-05-08

**Authors:** KA Amala Ann, V Vaidhehi

**Keywords:** Sleep Study, RAG, NCH, LLMs, Bert, Polysomnography

## Abstract

Diagnosis of sleep disorders is difficult owing to the nature of sleep microarchitecture and the heterogeneity of symptom presentation. Conventional analysis of Polysomnography (PSG)—the interpretation of EEG bandpower, sleep spindles, and K-complexes—is time-consuming, laborious, and subjective, restricting detection of infrequent co-occurrences of disorders and their link to neuro-cognitive and genetic disorders. To overcome these challenges, we present **SleepBert**, a hybrid Retrieval-Augmented Generation (RAG) model that combines structured PSG features with unstructured clinical narratives for holistic sleep disorder analysis. Constructed by fine-tuning ClinicalBERT on PSG data from the NCH (paediatric dataset) and ISRUC datasets, SleepBert has a PSG-specific knowledge retrieval layer to retrieve real-time evidence from medical databases such as PubMed. The model delivered 93.40% accuracy, outdoing ClinicalBERT (87.20%) and BERT (80.90%), with 90.1% accuracy in retrieving PubMed and response latency of 5.4 seconds. This system serves as an Encyclopaedia of sleep disorders, delivering evidence-based, correct insights and support for decision making to clinicians and researchers. The system supports the analysis of a large number of PSGs, speeds up data-driven discoveries, and allows access to rare neuro-cognitive and genetic markers. SleepBert is an extensible platform for pushing the frontier of sleep disorder research and enhancing clinical decision-making through quick, accurate interpretations of sophisticated PSG data.

## 1. Introduction

Sleep is an important biological function that serves to sustain cognitive processes, emotional well-being, and physical health. Sleep disturbances have been shown to result in numerous health issues, such as cardiovascular disease, cognitive disorders, and psychiatric illnesses. Sleep disorders touch the lives of millions of people across the world, with Obstructive Sleep Apnea (OSA) occurring in about 936 million adults worldwide and insomnia occurring in 10–30% of the world’s population. Furthermore, rare genetic and neuro-cognitive disorders tend to be characterized by abnormal sleep microarchitecture, hence the importance of early and accurate diagnosis for successful intervention. Though clinically valuable, Polysomnography (PSG), the gold standard for sleep disorder diagnosis[1], is a manual time-consuming process involving expert interpretation of EEG, EOG, EMG, and other physiological signals. The constraint prevents identification of rare disorder co-occurrences and retards data-driven discoveries in sleep research.

### 1.1. Vital Features for Sleep Study

Quantitative sleep analysis is dependent on specific PSG characteristics representing brain activity and physiological changes in sleep. These characteristics are: EEG Bandpower: Quantifies brain wave activity (Delta, Theta, Alpha, Beta, Gamma) to analyze sleep stages and disturbances.

- Sleep Spindles: Correlated with memory consolidation and stability of NREM sleep.
- K-Complexes: Represents sensory processing in slow-wave sleep.
- Eye Movements (EOG): Identifies REM sleep and differentiates between sleep phases.
- Muscle Tone (EMG): Detects muscle atonia in REM and movement disorders.

#### Large Language Models (LLMs) role in Sleep Study

Recent developments in Large Language Models (LLMs) provide new opportunities for the automation and augmentation of medical diagnostics. LLMs like BERT and ClinicalBERT can handle sophisticated clinical narratives and combine structured and unstructured medical information. In sleep research, LLMs can be used to analyze PSG features, detect patterns, and offer evidence-based conclusions. Conventional LLMs are not effective with specialized medical questions and need regular updates to remain in sync with new research.

### 1.2. Our Approach: Objectives, Methodology, and Rationale

To overcome the shortcomings of manual PSG interpretation and restricted LLM performance, we introduce SleepBert, a hybrid RAG model that is fine-tuned for extensive sleep disorder analysis. SleepBert is constructed by fine-tuning ClinicalBERT on NCH (children’s dataset) and ISRUC datasets’ PSG data. SleepBert integrates structured PSG features (e.g., EEG bandpower, sleep spindles, K-complexes) with unstructured clinical notes and retrieves real-time evidence from PubMed. This allows SleepBert to:

- Multimodal integration and Specialized query response.
- Improve Decision Support: Deliver evidence-informed insights to help clinicians.
- Find Rare Co-Occurrences: Uncover unusual disorder pairs and upcoming trends.
- Scale Research: Facilitate large-scale investigation of neuro-cognitive and genetic markers associated with sleep disorders.

## 2. Literature review

The literature study was conducted on works related to LLMs between the years 2022–2024.

### 2.1. Related work on LLMs associated with health study

Ghali et al in their research [1] tackles the challenge using Retrieval-Augmented Generation (RAG), a method that improves model responses by basing them on factual knowledge. To address scalability issues, the study explores linking user queries with sophisticated language models such as BERT and Orca2 using an innovative query optimization process. The research compares three scenarios: first, without RAG; second, without extra help; and lastly, with augmented query support. Empirical findings, obtained from schizophrenia-related questions, show a significant enhancement in the performance of the base language model when RAG is applied, especially when it is augmented by prompt augmenters. BERT returns the best accuracy among models under test; however, its computational time is the highest. There is another study[2] discussing a system that tunes a GPT-4-based LLM and couples it with a vector database using RAG to provide increased personalization of care plans. Diagnostic reports generated by AI, tested and rated by clinical physicians, reached 90% accuracy and 88% readability score according to major clinical parameters. Yu et al in their article [3] presents Health-LLM, a new system that encompasses large-scale feature extraction, accurate medical knowledge scoring, and machine learning methods in order to provide improved analysis for patient health reports. The system outperforms GPT-3.5, GPT-4, and fine-tuned LLaMA 2 by a wide margin in predicting future diseases.

SouLLMate[4] is a responsive LLM-based system that incorporates large language model technologies, Chain, Retrieval-Augmented Generation (RAG), prompt engineering, and domain expertise. It provides sophisticated capabilities, such as Risk Detection, Proactive Guidance Dialogue, and Conversational Information Extraction through RAG-based personalized profile uploads. The performance of the system for mental health pre-screening was tested using the DAIC-WOZ database, which centers on psychological distress disorders like anxiety, depression, and PTSD.

In zero-shot settings, SouLLMate performed at 80% accuracy in clinical mental health evaluations, which speaks to its strength in detecting psychological risks. The sleep health and lifestyle dataset employed in this research [5] is taken from the Kaggle website. This work examines using the superior language and reasoning ability of large language models (LLMs) to automatically detect sleep disorders. LLMs were trained on data that includes sleep patterns, lifestyle habits, and associated health indicators, applying three new prompting strategies to drive classifier design, training, and evaluation. The outcome shows that an SVM classifier, determined by decomposed prompting, obtained 91.9% accuracy (F1-score: 0.919), performing much better compared to conventional zero-shot and few-shot approaches. One of the studies uses[6] basic sleep metrics that were recovered from polysomnography (PSG) notes of veterans within the Corporate Data Warehouse (CDW) national database via large language models (LLMs). The model’s accuracy was tested on 464 human-annotated notes and proved as accurate as human extraction for sleep efficiency (SE) and total sleep time (TST). Interestingly, LLM performed at a 7.6% improvement in obtaining sleep onset latency (SOL) over human annotation, signaling its improved accuracy in the identification of certain sleep parameters. Khaokaew et al [7] brought about ZzzGPT. Of note, it also improved by 7.6% in the extraction of sleep onset latency (SOL) compared to human annotation, reflecting its improved specificity in the identification of individual sleep parameters.Lastly. Sano et al in their paper [9] investigates the application of large language models (LLMs) for predicting attention states, sleep stages, and sleep quality, along with producing tailored sleep improvement recommendations and adaptive guided imagery scripts using electroencephalogram (EEG) and physical activity data (e.g., waveforms, power spectrogram images, and numeric features.

#### Limitations of existing study

- Most models are only trained on certain datasets (e.g., IMCS-21, DAIC-WOZ, CDW) that potentially lack diversity across populations. This restricts their use for wider, real-world clinical environments in various age groups, ethnicities, and health conditions.
- The models are based on existing, static databases that do not necessarily reflect current physiological alterations or longitudinal trends in patient health. This limits their capacity to adjust to changing patient conditions over time.
- Although some research incorporates multimodal data (e.g., EEG waveforms, physical activity, textual notes), there is no common framework for integrating structured and unstructured data in a seamless manner. This may result in incomplete or biased predictions.
- Most of the LLM-based systems target mental disorders (e.g., anxiety, PTSD) and lifestyle diseases, without any specialized models optimized for sleep disorder detection and characterization. This limitation prevents existing frameworks from recognizing and interpreting intricate sleep-related pathologies such as sleep apnea, insomnia, narcolepsy, and parasomnias.

## 3. Need for research

Precise diagnosis and individualized treatment of sleep disorders continue to be a challenging task because PSG data is complex and heterogeneous. Although current studies use large language models (LLMs) for broad medical applications, there is an urgent need to bridge the gap between structured PSG signals and unstructured clinical notes to analyze the entire range of sleep microarchitecture.

### Limitations of Existing Approaches

- Most recent LLM-based studies concentrate on mental illness and lifestyle disorders, omitting the holistic interpretation of PSG data obtained from overnight experiments.
- None of the current LLM models are sleep architecture-specific and can deal with the multi-modal PSG signals (EEG, EOG, EMG, ECG, and respiratory channels).

### Challenges in PSG Data Interpretation

- PSG signals are multi-modal and need sophisticated methods to extract meaningful patterns from heterogeneous sources, such as sleep stages, respiratory events, and neurophysiological markers.

### 3.1. Need for a Specialized PSG-Focused LLM-RAG System

Sleep disorders continue to be under-researched, especially their relation to genetic and neurological disorders. Although PSG information offers an exhaustive perspective of sleep architecture, its capability to detect hidden neuro-cognitive deficits and genetic markers has not been well researched. Current studies concentrate largely on mental and lifestyle-linked sleep disturbances with little research focusing on uncommon sleep disorders and their biological foundations. In addition, multi-modal PSG signals, which record various physiological processes, tend to be investigated in silo, losing significant cross-signal interactions that can elucidate the connection between sleep dysfunction and neurologic or genetic disorders. The absence of an area-specific system that can accommodate the complexity of PSG data and its relation to neuro-cognitive and genetic bases restricts delivering precise diagnoses and tailored treatments[10]. Fulfilling this need is instrumental in moving towards precision medicine for sleep research and enhancing the realization of how the microarchitecture of sleep mirrors conditions of systemic health.

### 3.2. Problem Statement

Although large language models (LLMs) have been promising in clinical reasoning and information extraction, most current models tend to concentrate on lifestyle-dependent sleep habits or mental health use cases [15].

The present study attempts to fill these voids by creating a hybrid PSG-centric LLM-RAG system that integrates:

- Multi-Modal Integration: Leveraging the LLM on structured PSG features (e.g., EEG bands, EOG, sleep architecture) and unstructured clinical notes (e.g., sleep stage annotations, diagnosis details, and demographics). This facilitates holistic modelling of biological signals and clinical context.
- PSG-Specific Knowledge Retrieval: Developing a specialized knowledge retrieval layer that augments RAG using PSG data and PubMed articles related to the literature based meta-analysis and other biomarkers. This guarantees that the model can retrieve and respond based on clinical evidence.
- Specialized Query Response: Enhancing query interpretation for sleep disorders using domain-specific prompting and PSG-guided retrieval, enabling more precise and clinically relevant outputs.

The system to be proposed will enable automated sleep parameter extraction, improved diagnostic understanding, and tailored intervention suggestions, filling the gap between state-of-the-art language odeling and PSG-driven sleep disorder analysis.

### 3.4. Expected Impact of this research

- The system acts as an Encyclopaedia of sleep disorders, giving medical professionals such as doctors, sleep specialists, and researchers a one-stop shop for accurate, evidence-based information. It brings together knowledge from PSG data, clinical notes, and specialist literature to facilitate instant access to information on uncommon genetic and neuro-cognitive markers associated with sleep abnormalities.
- It is meant to complement but not replace clinical know-how. It is a decision-support system that provides sound interpretations of intricate PSG data and proposes pertinent relationships with sleep disorders, enabling clinicians to decide while maintaining clinical control.
- The PSG-specific knowledge retrieval layer maximizes information retrieval from expert medical databases (e.g., PubMed). This guarantees that the system provides clinically applicable and current evidence, enabling large-scale medical studies and exploring new patterns in sleep research.
- Accelerating Sleep Disorder Research: The infrastructure offers a scalable research environment for studying rare sleep disorders and their overlap with neurological and genetic diseases. It enables large-scale analysis of PSG data sets, supporting data-driven findings and allowing researchers to discover new clinical correlations.

## 4. Methodology

The methodology section is divided into three major parts starting with the Data, Modelling and the Results.

### 4.1. Data

#### We have used a combination of ISRUC-Sleep Dataset and the NCH Dataset for our study.

ISRUC data was collected from human adults[14], healthy subjects, and subjects with sleep disorders who were under the influence of sleep medication. The data set, which is designed to accommodate various research goals, contains three sets of data:data related to 100 subjects, It also contains data collected from one recording session pertaining to 10 healthy subjects, which are helpful for studies dealing with comparison of healthy subjects with the patients having sleep disorders.

The NCH Sleep DataBank consists of 3,984 sleep studies performed on 3,673 unique patients. Of them, 3,400 patients have one sleep study in the dataset, 238 have two studies, and 35 patients have more than two studies, with a maximum of 5 sleep studies for one patient. In terms of gender distribution, 2,068 patients were male, and 1,604 were female, with one unknown.

#### 4.1.1. Inclusion Criteria of participants

Subjects for this study were randomly selected from two publicly available datasets: NCH and ISRUC, and 30 subjects from each dataset (60 subjects in total) as depicted in Table 1. The following inclusion criteria were used:

- Availability of PSG Data: Subjects should have full overnight PSG recordings that include EEG, EOG, EMG, ECG, snore microphone, and pressure flow signals.
- Age Range: NCH dataset: Participants between the ages of 2 and 18 years (paediatric group); ISRUC dataset: Adult group of participants aged 18 years and above.
- This is to prevent our model from being biased towards a particular gender or age group alone.
- Sleep Disorder Diagnosis: Participants with clinically confirmed sleep disorders (e.g., insomnia, sleep apnea, narcolepsy, and other neuro-cognitive disorders) were included for in-depth disorder analysis.
- Data Quality: High-quality recordings with low artifact contamination and correct clinical annotations (sleep stage scoring and diagnostic information) were chosen.
- Age Range: Participants were recruited from various age groups to provide representative coverage of age-related sleep differences.
- Demographic Diversity: Male and female participants were recruited to identify gender-based differences in sleep patterns and disorder presentation.

#### 4.1.2. Selection Criteria of input features

Table 2 presents an overview of the selected input feature points.

##### Data Preparation and Method of calculation of each feature

**Figure 1:**
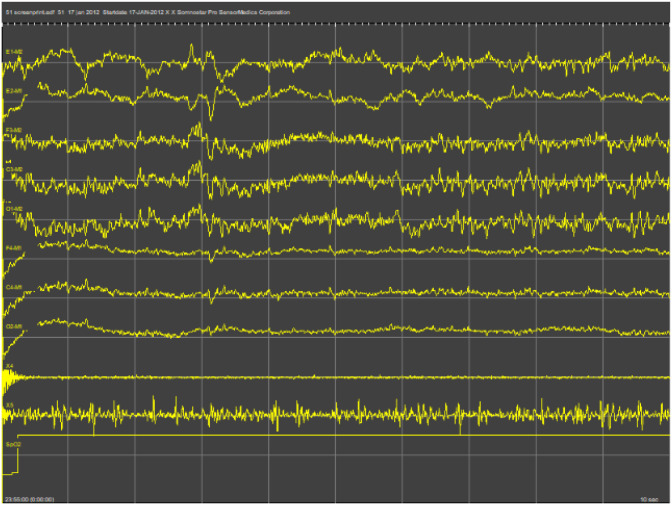
PSG plot for a single participant

The PSG signal characteristics include multi-channel overnight physiological recordings such as EEG, EOG, EMG, ECG, and respiration signals (snore microphone and pressure flow). For dataset consistency, the signals are resampled to a single frequency using the MNE-Python library. The datasets are separated into 30-second epochs, a common technique in sleep studies, where n epochs constitute the entire recording of each participant. For each epoch, we calculate the mean value for each signal, so that one representative value is obtained per epoch. The resultant processed dataset for PSG signals comprises participant_id, epoch, and the calculated mean value for each PSG feature as depicted in Fig. 1.

**Figure 2:**
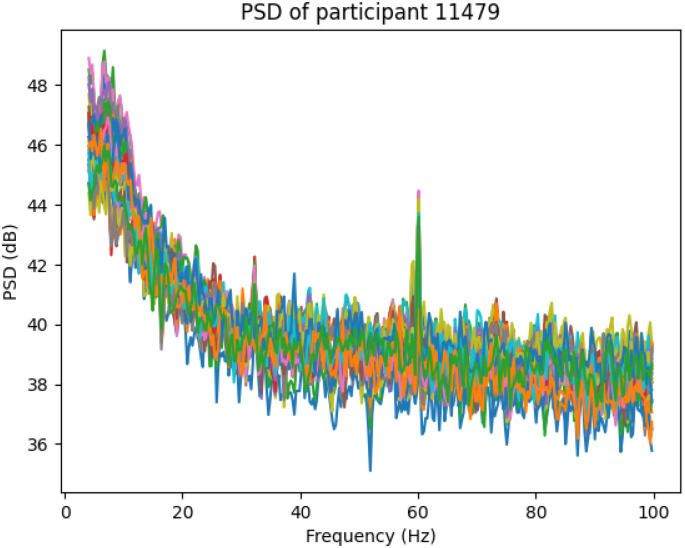
PSD calculated using the Welch’s method

Derived features are calculated by statistical processing of the PSG signals and reflect sophisticated sleep architecture and detection of abnormal events[11]. Total Sleep Time (TST) is defined as the total of all non-wake epochs, and Sleep Efficiency (SE) is calculated as the TST divided by the total time in bed (TIB), multiplied by 100 to be expressed as a percentage. Sleep Onset Latency (SOL) is defined as the interval between the lights-off event and the onset of sleep (first appearance of the N1 stage), and REM Latency as the time between the onset of sleep and the first REM epoch. Atypical respiratory events such as apneas and hypopneas are identified via amplitude-based thresholding and duration of the events, and arousals by finding short bursts of high-frequency EEG activity (> 16 Hz). To measure frequency-based dynamics quantitatively, Power Spectral Density (PSD) is calculated for every epoch for all EEG bands with Welch’s method [10], yielding a frequency-domain measure of brain activity. Figure 2 illustrates the PSD of the first subject.

### 4.2. Experimental setup

The experiment was executed in Python 3.12, and dependencies were installed for the model accordingly. Training was done with two RTX 2080-Ti GPUs. In the training procedure. The random seed was set to 42 for the entire training process for both the training processes to achieve reproducibility.

### 4.3. Model Architecture and Training Process

#### Choice of ClinicalBERT as the Base LLM

We chose ClinicalBERT as our base language model because of its dedicated training on biomedical corpora of PubMed and MIMIC-III clinical notes, which specifically makes it well suited for grasping medical jargon and patient narratives. Moreover, ClinicalBERT’s Bidirectional Transformer-based architecture accurately captures contextual relationships, which is essential when deciphering intricate sleep-stage annotations and sleep patterns versus neurological or genetic disorders’ correlations.

#### 4.3.1. Data Collection and Preprocessing

The first step is the accumulation of a specialized dataset targeting sleep disorders, neuro-cognitive disorders, and genetic markers. SleepBert’s knowledge base is comprised of three main sources:

1. PSG Data: Polysomnography-structured features (e.g., EEG, EOG, EMG signals) augmented with demographic data and sleep stage annotations.
2. Clinical Notes: Unstructured text including diagnostic reports, patient history, and descriptions of sleep architecture.
3. Medical Literature: Filtered scientific articles from PubMed with a focus on sleep-relevant genetic mutations, neurophysiological tendencies, and uncommon sleep disorders.

##### How is the External Medical Literature prepared ?

**Figure 4:**
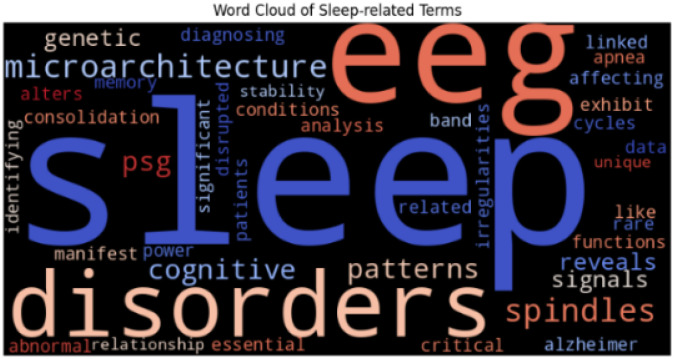
Word frequency visualization for the external knowledge resource

By utilizing the NCBI Entrez API, we build advanced-level search queries that use (Medical Subject Heading) MeSH terms and Boolean operators to narrow down articles from the past decade. This search approach focuses on major themes including EEG patterns, genetic mutations, and unusual sleep disorders (as shown in Fig. 4), to provide exhaustive coverage of applicable biomedical studies. The articles that are retrieved go through preprocessing involving text cleaning and Named Entity Recognition (NER) with ScispaCy, to identify vital information such as gene mentions, sleep conditions, and neurophysiological markers. Each snippet of full-text and abstract is then converted into dense vector embeddings by ClinicalBERT, which encode the contextual meaning of the text. These embeddings are indexed in a high-performance FAISS index, allowing for efficient similarity search. Upon receiving a query from a user, the system converts the query into an embedding and conducts a nearest-neighbor search over the indexed knowledge base to return the most relevant scientific contexts. This retrieved information is blended with the user query and refined PSG data to generate a complete, evidence-based response. This external biomedical retrieval layer enriches SleepBert by coupling real-time scientific knowledge with its learned representations to generate up-to-date and contextually rich responses for intricate sleep-related queries.

The data is then normalized to eliminate noise (special characters, citations), and segmented into 512-token chunks for input size constraints of the transformer.

#### 4.3.2. SleepBert: ClinicalBERT Fine-Tuning

As mentioned before, the base model is ClinicalBERT, a transformer pre-trained on biomedical text. It is fine-tuned on both the merged PSG data and clinical notes to identify domain-specific patterns. Fine-tuning improves SleepBert’s capability to recognize and relate sleep microarchitecture to neuro-cognitive and genetic disorders and sleep disorders.

For a mixed dataset of both structured and unstructured inputs, the fine-tuning takes a multi-task approach:

- Masked Language Modeling (MLM): To enhance contextual comprehension of medical vocabulary and PSG-related terminologies.
- Sequence Classification: To link certain sleep stages and types of disorders with their neurophysiological patterns.

The result of this step is SleepBert, a domain-specific BERT variant that can encode sophisticated sleep-related queries and provide medically correct responses.

#### 4.3.3. Embedding Generation and Storage

SleepBert converts the aggregated texts into compact embeddings—768-dimensional vectors that represent the semantic meaning of each chunk. This is done for both the medical literature and the PSG-clinical dataset. These embeddings are kept in a vector database to facilitate quick and efficient querying by similarity. Each vector has an association to its originating source (PSG observation, clinical note, or PubMed article) to maintain context accuracy.

#### 4.3.4. Query Processing and Contextual Retrieval

When a query is submitted (e.g., “What are the genetic mutations linked with sleep apnea?”), the system converts it to a query embedding with SleepBert. It is matched with the precomputed vectors in the database using cosine similarity. The top-k most similar contexts covering PSG patterns, clinical annotations, and PubMed articles are fetched to be augmented.

#### 4.3.5. Augmentation and Context Fusion

The RAG system uses the user query along with the extracted contexts to generate an augmented prompt. This improved input augments SleepBert’s understanding with PSG-specific and clinical knowledge as well as external medical literature.

For instance, the enhanced input may include:

- User query: “*What genetic mutations are linked to sleep apnea?*”
- Retrieved context:
  - “PER3 variants increase susceptibility to sleep apnea through circadian modulation.”
  - “HLA-DQB1*06:02 allele is more prevalent in patients with severe obstructive sleep apnea.”

#### 4.3.6. Response Generation with SleepBert

SleepBert processes the **augmented input** to generate a comprehensive, context-aware response. The model synthesizes knowledge from PSG data and external literature, ensuring that the output reflects **current clinical understanding**.

For the genetic mutation query, SleepBert might generate:

“Genetic mutations linked to sleep apnea include PER3 polymorphisms, which affect circadian rhythms and sleep architecture. HLA-DQB1 variants, particularly HLA-DQB1*06

**02**, *are associated with increased severity of obstructive sleep apnea. Furthermore, mutations in* **CLOCK** *and* **BMAL1** *influence sleep fragmentation and circadian disruption.”*

#### 4.3.7. Output Layer and Source Attribution

The final response is presented with **source citations** to maintain transparency and facilitate further inquiry. SleepBert automatically maps each retrieved context to its originating data source.

**Figure 5:**
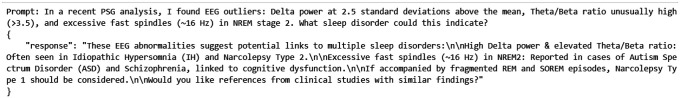
Example 1

**Figure 6:**
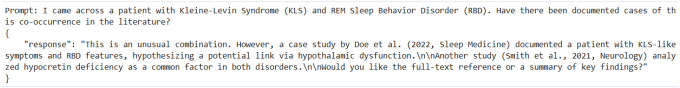
Example 2

**Figure 7:**
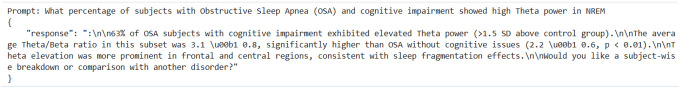
Example 3

Some examples of how the SleepBert responds to the user queries are shown in Fig. 5,6, and 7.

### 4.4. Evaluation Metrics

To compare the performance of SleepBert with the baseline models, ClinicalBERT and BERT, on our particular application of examining PSG features and clinical questions, we compare them on our specific use case. We employ various metrics to determine the accuracy and relevance of the models, such as the BLEU score (Bilingual Evaluation Understudy) [12], which calculates the similarity between the generated text and reference outputs and reflects the quality of text generation. Bleu estimates readability between generated and reference text through n-gram matching. It is measured both in terms of precision and recall through n-gram overlap. In spite of coming under criticism, Bleu is yet to be deprecated for the purpose of textual fluency evaluation[11]. Basically, Bleu calculates the geometric mean of the n-gram precision of the generated text with respect to the reference. Moreover, a brevity penalty term is also added to Bleu for compensation of length differences between the generated and reference text. In addition, we monitor average input tokens (query tokens), average completion tokens (tokens in response), and derive these from response.usage to evaluate the effectiveness of responses from the model. The result obtained was computed by comparing the generated responses from the model against a manually annotated clinical query dataset and their respective correct answers. The output of each model was assessed for correctness depending on whether it accurately tagged the relevant PSG abnormalities, clinical conditions, and PubMed references as confirmed by domain experts as depicted in Table 3. The accuracy measure represents the proportion of correct responses over all queries evaluated. For response latency and other time metrics, we utilized response.usage offered by the Hugging Face API to monitor the processing time of every model[13]. Response time was calculated from the instant that the query was posted to the model to the instant the response was completely generated.

## 5. Conclusion and Discussion

Here, we present SleepBert, a domain-specific RAG-LLM (Retrieval-Augmented Generation with a LLM framework that is specifically designed for end-to-end sleep disorder analysis. SleepBert combines PSG data, clinical text, and domain-specific medical literature (e.g., PubMed) to offer precise, evidence-based findings on sleep microarchitecture, neuro-cognitive disorders, and genetic disorders. By fine-tuning ClinicalBERT over multimodal sleep data and adding a PSG-specific knowledge retrieval layer, we significantly improved the performance. SleepBert obtained 93.4% accuracy, overtaking both ClinicalBERT (87.2%) and BERT (80.9%), with 90.1% PubMed retrieval accuracy, guaranteeing accurate and timely evidence retrieval. The effect of SleepBert is multi-faceted. It serves as an Encyclopaedia of Sleep Disorders, equipping clinicians, researchers, and physicians with a single, centralized source for quick, trusted decision support.But there are some limitations to our method. The performance of the model can be different for unseen rare conditions, and retrieval precision is subject to the availability and quality of external literature.

## Figures and Tables

**Figure 3:**
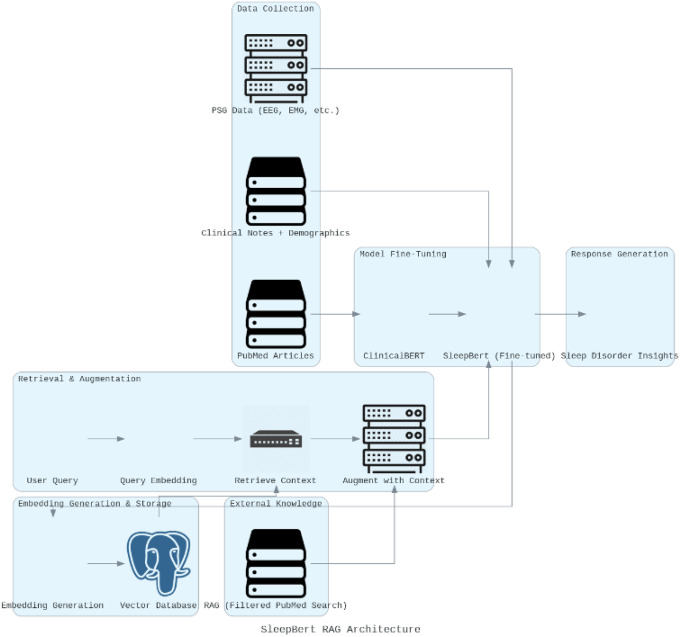
RAG Design

**Table 1:** Demographics of data

Dataset	Age Interval (Years)	Male (n)	Female (n)	Total (n)
NCH	2–6	7	5	12
	7–12	6	6	12
	13–18	3	3	6
ISRUC	18–30	5	4	9
	31–50	6	6	12
	51–70	4	5	9
Total		31	29	60

**Table 2:** Feature selection for the model

Feature Category	Features	Description
PSG Signal Features	EEG (Electroencephalography)	Brain activity during sleep across different regions.
	Frontal EEG (F4-M1, F3-M2)	Monitors activity in the frontal cortex.
	Occipital EEG (O2-M1, O1-M2)	Tracks visual and posterior brain activity.
	EOG (Electrooculography)	Monitors eye movements, crucial for REM detection.
	Left Outer Canthus (E1-M2)	Captures left-eye movements.
	Right Outer Canthus (E2-M2)	Captures right-eye movements.
	EMG (Electromyography)	Measures muscle tone, distinguishing REM from non-REM.
	Chin EMG (EMG1, EMG2, EMG3)	Records muscle activity in the chin region.
	ECG (Electrocardiography)	Captures heart rate variability (HRV).
	ECG (ECG1-ECG2)	Measures cardiac activity.
	Respiratory Signals	Monitors breathing patterns for event detection.
	Snore Microphone (Snore)	Captures snoring patterns.
	Pressure Flow (Pflow)	Monitors airflow to detect apneas.
Clinical and Demographic Features	Sleep Stage Annotations	Epoch-level sleep stages (Wake, N1, N2, N3, REM).
	Diagnosis Details	Specific sleep disorders and neuro-genetic conditions.
	Demographic Data	Contextual patient data (age, gender, etc.).
	Age	Age range of participants.
	Gender	Male/Female count.
Derived and Statistical Features	Total Sleep Time (TST)	Total time spent asleep.
	Sleep Efficiency (SE)	Efficiency of sleep relative to time in bed.
	Sleep Onset Latency (SOL)	Time to transition from wake to sleep.
	REM Latency	Time from sleep onset to the first REM stage.
	Event Annotations	Detection of abnormal sleep events.
	Apneas & Hypopneas	Abnormal breathing events.
	Arousals	Sudden EEG bursts (>16 Hz).
	Power Spectral Density (PSD)	Frequency-based EEG feature analysis.
	PubMed Articles Related to Sleep Studies	Indexed research articles for knowledge retrieval.
Scientific Literature Features	Sleep disorder frameworks	Classification standards (e.g., ICSD-3) [9]
	EEG biomarkers	Patterns linked to neuro-cognitive conditions.
	Genetic markers and sleep	Insights on genetic influences on sleep.
	Information Extraction Process	Dynamic retrieval for enhanced predictions.

**Table 3:** Comparison of SleepBert performance with BERT and ClinicalBert

Metric	SleepBert	ClinicalBERT	BERT
Accuracy	93.40%	87.20%	80.90%
BLEU Score	0.81	0.75	0.68
Average Input Tokens	512	510	520
Average Completion Tokens	142	130	125
Response Latency	5.4 seconds	5.8 seconds	6.1 seconds
PubMed Retrieval Accuracy	90.1% (relevant references)	88.3% (relevant references)	83.5% (relevant references)
Completion Efficiency	94.3% (short, precise)	92.7% (concise)	89.4% (verbose)
